# Conducting human challenge studies in LMICs: A survey of researchers and ethics committee members in Thailand

**DOI:** 10.1371/journal.pone.0223619

**Published:** 2019-10-10

**Authors:** Jaranit Kaewkungwal, Pornpimon Adams, Jetsumon Sattabongkot, Reidar K. Lie, David Wendler

**Affiliations:** 1 Department of Tropical Hygiene, Faculty of Tropical Medicine, Mahidol University, Bangkok, Thailand; 2 Office of Research Services, Faculty of Tropical Medicine, Mahidol University, Bangkok, Thailand; 3 Mahidol Vivax Research Unit, Faculty of Tropical Medicine, Mahidol University, Bangkok, Thailand; 4 Department of Philosophy, University of Bergen, Bergen, Norway; 5 Department of Bioethics, National Institutes of Health Clinical Center, National Institutes of Health, Bethesda, Maryland, United States of America; University Medical Center Utrecht, NETHERLANDS

## Abstract

Questions have been raised over the acceptability of conducting human challenge studies in low and middle income countries (LMICs). Most of these concerns are based on theoretical considerations and there exists little data on the attitudes of stakeholders in these countries. This study examines the view of researchers and REC members in Thailand regarding the design and conduct of challenge studies in the country. A questionnaire was developed based on ethical frameworks for human challenge studies. The target respondents included those who had experience with health-related research at universities, non-university hospitals, and research institutes. A total of 240 respondents completed the on-line survey. In general, the respondents felt that the ethical issues raised by human challenge studies in LMICS do not differ significantly from those in high income countries, including: scientific rationale, safety, appropriate risks, and robust informed consent process. In contrast, issues that have been described as important for human challenge studies in LMICs were rated as having lower importance, including: a publicly available rationale, national priority, and community engagement. Responses did not vary significantly between researchers in different fields, nor between researchers and REC members. These findings provide an important perspective for assessing existing frameworks for human challenges studies in LMICs.

## Introduction

Human challenge studies involve the intentional infection of study participants with disease-causing pathogens as a means to assess potential vaccines or drug candidates, or to understand how humans are protected from or acquire the infection [1–5]. Many commentators agree that such studies may be approved when they satisfy a number of requirements, including: a highly controlled environment, robust informed consent, and effective treatment for the infection [6]. Permitting human challenge studies under these conditions is consistent with the more general view that it can be permissible to expose research participants to greater than minimal risk when the study is valuable, the risks are not excessive, participants give informed consent, and participants are compensated for any research harms [3,4,6].

To date, most human challenge studies have been conducted in high income countries. With technological advancement, well-established laboratory facilities and increasing capacity, some have proposed that challenge studies might also be conducted in low and middle income countries (LMICs) [7–13]. Others express concern over whether these studies would be appropriate. In particular, questions have been raised over the safety of participants and the availability of suitable care for (infected) participants in LMICs. Further concerns expressed are whether the public in LMICs would accept human challenge studies and the possibility that participants might be unduly induced to participate by offers of (excessive) payment [14].

These concerns over conducting human challenge studies in LMICs are based on theoretical considerations and there exists little data on the attitudes of stakeholders in these countries. The present study was designed to address this gap in the literature. Thailand, as one of the LMICs, has conducted a few human challenge studies including, for example, trials on controlled human malaria infection, oral inoculation with *Vibrio cholerae*, and *Shigella sonnei* vaccine [7–9]. Specifically, this study examines the view of researchers and REC members in Thailand. Do they believe that conducting human challenge studies in Thailand would be appropriate? What do they regard as the most important concerns raised by these studies?

## Materials and methods

### Target study respondents

The target respondents were REC members and researchers who had experience with health-related research at universities, non-university hospitals, and research institutes. We attempted to identify respondents from a range of fields, including biomedicine, pharmacology, clinical practice, epidemiology and public health, and behavioral-social science,

### Data collection

Paper-based and online versions of the survey were developed. The on-line survey was distributed via e-mails that contained a link to the questionnaire. The paper-based and online versions of the survey were distributed to 218 participants from 38 academic and health-science institutes across Thailand who were participating in a 2018 workshop on human research studies organized by the Office of Research Services of the Faculty of Tropical Medicine (FTM), Mahidol University. The online version was subsequently sent to the heads of the research offices at 18 university hospitals, 84 non-university hospitals, and 22 research institutes, as well as alumni and researchers who had previously submitted proposals to and/or participated in workshops conducted by FTM. In total, 2,656 emails were sent from FTM. In addition, recipients of the on-line survey were asked to forward it to colleagues in their field.

Recipients were informed that completing the survey was voluntary. The survey was anonymous, and not linked to the submitting source. Completed surveys were uploaded automatically to a database.

### Questionnaire development

#### Step 1. Identifying issues raised by challenge studies

A review of international and local ethics guidelines, as well as discussions in scientific journals, was used to identify the important ethical issues related to designing and conducting human challenge studies. This search identified a number of frameworks and best practices [3–6]. One framework was based on interviews with experts on challenge studies. This framework focuses on: ensuring volunteer safety; assessing the potential for transmission to third parties; considering the potential public health impact; ensuring there are no alternative means to gather the same information; and collecting reliable and relevant data [15].

A second framework was developed by a committee consisting of US federal employees, researchers, and ethicists organized by the National Institute of Allergy and Infectious Diseases (NIAID) and the Walter Reed Army Institute of Research (WRAIR). This framework discusses 8 issues relevant to challenge trials involving Zika virus: (1) minimizing risks and ensuring the remaining risks are justified by the potential social value of the study; (2) protecting vulnerable populations; (3) ensuring informed consent process; (4) offering adequate but not undue levels of compensation; (5) guaranteeing the right to withdraw;(6) obtaining independent expert review; (7) providing compensation for research injury; and (8) obtaining community engagement [15].

A third framework describes 5 common criteria for any trial involving human subjects and 4 criteria specific to human challenge studies [6]. The five common criteria are: (1) potential to collect important scientific knowledge; (2) absence of satisfactory alternative methods; (3) robust informed consent from competent adult volunteers; (4) acceptable risk-benefit ratio; and (5) equitable selection of study participants. The four criteria specific to challenge trials are: (6) independent expert/systematic review; (7) publicly available rationale and protection of public confidence; (8) protection of the public from the infection; and (9) compensation for any research harms.

A final framework for potential human challenge studies in Malawi addressed 7 major issues: (1) importance of informed consent, (2) value of the study for Malawi; (3) capacity development in the country; (4) strong scientific basis for the study, with no feasible alternatives, (5) appropriate design based on the published literature; (6) governance (e.g., DSMB, sponsor); and (7) safety of the pathogen [3].

The questionnaire used for the present study was based on these frameworks as well as the expanded framework for the ethical evaluation of human challenge studies developed by Emerson and Cullen [16]. Based on this work, we identified the following sixteen items as potential concerns raised by human challenge studies in general: scientific rationale, absence of alternatives, technical considerations, independent review, informed consent, risks and harms, balance of risks and benefits, safety, selection of participants, compensation for harms, publicly available rationale, protection of the public, knowledge and data sharing, community engagement, governance, and national priority. We further identified the following 9 as potential concerns for conducting human challenge studies in LMICs: Quality assurance / quality control; Good manufacturing practices; Appropriate compensation; Community perception and engagement; Sponsorship and intellectual property rights; Readiness of infrastructure; National priority; Community acceptance; Vulnerable subjects (S1 File)

Respondents were asked to rate the importance of each issue on a Likert scale from 1–5, with 1 indicating least important and 5 indicating most important.

#### Step 2. Determining the validity of the questionnaire items

The questionnaire was developed in both English and Thai, as the respondents included Thai and non-Thai researchers in different organizations. Three REC members of the Faculty of Tropical Medicine reviewed a draft of the questionnaire for face validity. The dual-language questionnaire was cross-validated by a native English speaker.

#### Step 3. Test of the online questionnaire

No formal pilot study of the final questionnaire was performed. Instead, the paper-based and on-line versions were distributed to participants in the workshop who were asked to provide feedback. The workshop participants did not identify any significant issues, so the questionnaire was considered acceptable.

### Data analysis

We assumed that responses regarding the importance of the listed items would skew toward the upper range of the scale. Thus, we collapsed the 5-point scale into 3 categories: 5 = very important, 4 = important, and 1–3 = less important. We then calculated a frequency and percentage by respondent characteristics: clinical and non-clinical researchers, and researchers and REC members. Rating level comparisons were evaluated using chi-square tests, with a p-value of <0.05 considered statistically significant.

### Ethical clearance

This project was approved by The Ethics Committee of the Faculty of Tropical Medicine, Mahidol University, Thailand, with Certificate of Approval Number MUTM-EXMPT 2018–003. The Ethics Committee certified that the study protocol was in compliance with the Declaration of Helsinki, ICH Guidelines for Good clinical Practice, and other international guidelines for human research protection. We confirm that informed consent was obtained voluntarily from all participants. No identifiers could link to the identities of the questionnaire respondents. They could also decide not to answer any question in the questionnaire, without any negative impact.

## Results

### Characteristics of the survey respondents

A total of 240 respondents completed the survey (S2 File). Based on the 2,656 surveys known to have been distributed, this suggests an overall response rate of 9% (240/2,656). As shown in Table 1, the ratio of male to female respondents was 30:70. Half of the respondents worked mainly in clinical studies, while the other half worked in basic and applied sciences. Years of experience ranged from 1–3 years to > 15 years. About 30% of respondents reported current or previous experience as the member of an ethics committee.

**Table 1:** Characteristics of study respondents (N = 240).

Characteristics of respondents	n	%
Sex		
• Male	71	29.6
• Female	169	70.4
Main research field:		
• Clinical study	123	51.3
• Biomedical/laboratory study	60	25.0
• Public health/Policy research	17	7.1
• Social science/behavioral research	28	11.7
• Other	12	5.0
Years working in research field:		
• 1–3	48	20.4
• 4–6	32	13.6
• 7–10	33	14.0
• 1–15	36	15.3
• > 15	86	36.6
Experience on an ethics committee		
• No	164	68.3
• Yes	76	31.7

### Considerations regarding designing and conducting challenge studies

As expected, most of the items were rated as very important (5) or important (4). As shown in Table 2, >80% of respondents rated as very important: safety, informed consent, scientific rationale, risks and harms. The issues rated by >70% of respondents as very important were: governance, balance of risk-benefit, and protection of public. About 60–68% of respondents rated as very important: selection of study participants, compensation for harm and publicly available rationale. The issues that > 50–58% of respondents rated as very important included: knowledge and data sharing, technical considerations, independent review, absence of alternative, national priority, and community engagement.

**Table 2:** Ratings of important issues in designing and conducting challenge studies (N = 240).

Important Issues in Designing and Conducting Challenge Studies	Total (N = 240)		
	Very Important (5)	Important (4)	Less Important (1–3)
	n (%)	n (%)	n (%)
Safety	215 (89.6)	23 (9.6)	2 (0.8)
Informed consent	208 (86.7)	30 (12.5)	2 (0.8)
Scientific rationale	201 (83.8)	33 (13.7)	6 (2.5)
Risks and harms	198 (82.5)	37 (15.4)	5 (2.1)
Governance	187 (77.9)	46 (19.2)	7 (2.9)
Balance of risks and benefits	182 (75.8)	54 (22.5)	4 (1.7)
Protection of public	176 (73.3)	59 (24.6)	5 (2.1)
Selection of study participants	165 (68.7)	68 (28.3)	7 (2.9)
Compensation for harm	162 (67.5)	71 (29.6)	7 (2.9)
Publicly available rationale	150 (62.5)	85 (35.4)	5 (2.1)
Knowledge and data sharing	140 (58.3)	88 (36.7)	12 (5.0)
Technical considerations	139 (57.9)	88 (36.7)	13 (5.4)
Independent review	130 (54.2)	92 (38.3)	18 (7.5)
Absence of alternatives	128 (53.3)	85 (35.4)	27 (11.3)
National priority	122 (50.8)	95 (39.6)	23 (9.6)
Community engagement	121 (50.4)	93 (38.8)	26 (10.8)

The ratings of clinical and non-clinical researchers were similar to the overall ratings and we did not identify any statistically significant differences in their responses (Table 3). Respondents with present or previous experience as REC members were more likely to rate items as very important (5) compared to respondents who lacked experience as REC members (Table 4). These differences led to statistically significant differences between the two groups regarding the importance of: balance of risk-benefit (rated as very important by 80% of REC members vs. 74% of researchers, p-value = 0.05) and selection of study participants (rated as very important by 74% of REC members vs. 66% of researchers, p-value < 0.01).

**Table 3:** Comparisons of ratings about challenge studies by clinical and non-clinical researchers.

Issues in Designing and Conducting Challenge Studies	Clinical (N = 123)			Non-Clinical (N = 117)			p-value
	Very Important	Important	Less Important	Very Important	Important	Less Important	
	n (%)	n (%)	n (%)	n (%)	n (%)	n (%)	
Safety	113 (91.9)	8 (6.5)	2 (1.6)	102 (87.2)	15 (12.8)	0 (0.0)	0.10
Informed consent	108 (87.8)	13 (10.6)	2 (1.6)	100 (85.5)	17 (14.5)	0 (0.0)	0.26
Scientific rationale	101 (82.1)	18 (14.6)	4(3.3)	100 (85.5)	15 (12.8)	2 (1.7)	0.67
Risks and harms	103 (83.7)	17 (13.8)	3 (2.4)	95 (81.2)	20 (17.1)	2 (1.7)	0.74
Governance	98 (79.7)	23 (18.7)	2 (1.6)	89 (76.0)	23 (19.7)	5 (4.3)	0.46
Balance of risk-benefit	95 (77.2)	26 (21.1)	2 (1.6)	87 (74.4)	28 (23.9)	2 (1.7)	0.87
Protection of public	99 (73.1)	29 (23.6)	4 (3.3)	86 (73.5)	30 (25.6)	1 (0.9)	0.42
Selection of study participants	81 (65.9)	40 (32.5)	2 (1.6)	84 (71.8)	28 (23.9)	5 (4.3)	0.19
Compensation for harm	82 (66.7)	37 (30.0)	4 (3.3)	80 (68.4)	34 (29.0)	3 (2.6)	0.93
Publicly available rationale	78 (63.4)	42 (34.2)	3 (2.4)	72 (61.5)	43 (36.8)	2 (1.7)	0.86
Knowledge and data sharing	70 (56.9)	47 (38.2)	6 (4.9)	70 (59.8)	41 (35.0)	6 (5.1)	0.88
Technical considerations	70 (56.9)	48 (39.0)	5 (4.1)	69 (59.0)	40 (34.2)	8 (6.8)	0.53
Independent review	69 (56.1)	43 (35.0)	11 (8.9)	61 (52.1)	49 (41.9)	7 (6.0)	0.44
Absence of alternative	71 (51.7)	38 (30.9)	14 (11.4)	57 (48.7)	47 (40.2)	13 (11.1)	0.31
National priority	65 (52.8)	50 (40.7)	8 (6.5)	57 (48.7)	45 (38.5)	15 912.8)	0.25
Community engagement	56 (45.5)	53 (43.1)	14 (11.4)	65 (55.5)	40 (34.2)	12 (10.3)	0.29

**Table 4:** Comparisons of ratings about challenge studies by researchers and REC members.

Issues in Designing and Conducting Challenge Studies	REC members (N = 76)			Researchers (N = 164)			p-value
	Very Important	Important	Less Important	Very Important	Important	Less Important	
	n (%)	n (%)	n (%)	n (%)	n (%)	n (%)	
Safety	69 (90.8)	6 (7.9)	1 (1.3)	146 (89.0)	17 (10.4)	1 (0.6)	0.72
Informed consent	69 (90.8)	7 (9.2)	0 (0.)	139 (84.8)	23 (14.0)	2 (1.2)	0.35
Scientific rationale	63 (82.9)	11 (14.5)	2 (2.6)	138 (84.2)	22 (13.4)	4 (2.4)	0.97
Risks and harms	62 (81.6)	11 (14.5)	1 (3.9)	136 (82.9)	26 (15.9)	2 (1.2)	0.38
Governance	62 (81.6)	10 (13.2)	4 (5.2)	125 (76.2)	36 (22.0)	3 (1.8)	0.11
Balance of risk-benefit	61 (80.3)	12 (15.8)	3 (3.9)	121 (73.8)	42 (25.6)	1 (0.6)	0.05
Protection of public	54 (71.1)	20 (26.3)	2 (2.6)	122 (74.4)	39 (23.8)	3 (1.8)	0.83
Selection of study participants	56 (73.7)	14 (18.4)	6 (7.9)	109 (66.5)	54 (32.9)	1 (0.6)	<0.01
Compensation for harm	54 (71.1)	19 (25.0)	3 (3.9)	108 (65.9)	52 (31.7)	4 (2.4)	0.50
Publicly available rationale	46 (60.5)	28 (36.8)	2 (2.6)	104 (63.4)	57 (34.8)	3 (1.8)	0.86
Knowledge and data sharing	44 (57.9)	25 (32.9)	7 (9.2)	96 (58.5)	63 (38.4)	5 (3.1)	0.11
Technical considerations	44 (57.9)	30 (39.5)	2 (2.6)	95 (57.9)	58 (35.4)	11 (6.7)	0.40
Independent review	45 (59.2)	23 (39.3)	8 (10.5)	85 (51.8)	69 (42.1)	10 (6.1)	0.15
Absence of alternative	39 (51.3)	32 (42.1)	5 (6.6)	89 (54.3)	53 (32.3)	22 (13.4)	0.16
National priority	40 (52.6)	28 (36.9)	8 (10.5)	82 (50.0)	67 (40.9)	15 (9.1)	0.83
Community engagement	40 (52.6)	30 (39.5)	6 (7.9)	81 (49.4)	63 (38.4)	20 (12.2)	0.60

### Concerns about conducting challenge studies in Thailand

As shown in Table 5, about 40% of respondents rated as very important the quality assurance / quality control procedures of human challenge studies conducted in Thailand. About 30% rated as very important and 40% rated as important the following issues: appropriate compensation, community perception and engagement, and intellectual propriety rights of the finished product. Fewer respondents rated as very important readiness of infrastructure, national priority, community acceptance, and vulnerabilities pertaining to the informed consent process.

**Table 5:** Opinions regarding conducing challenge studies in Thailand.

Major Concerns in Conducting Challenge Studies in Thailand	Total (N = 240)		
	Very Important	Important	Less Important
	n (%)	n (%)	n (%)
Quality assurance / quality control procedures in the agent used in challenge study following Good Manufacturing Practice principles	104 (43.3)	82 (34.2)	54 (22.5)
Appropriate compensation for risk-taking	84 (35.0)	98 (40.8)	58 (24.2)
Community perception and engagement with respect to the understanding of infection risk, disease severity, treatment availability	83 (34.6)	98 (40.8)	59 (24.6)
Sponsorship and intellectual propriety rights of the finished product	72 (30.0)	97 (40.4)	71 (29.6)
Readiness of infrastructure and clinical facilities for challenge study	67 (27.9)	114 (47.5)	59 (24.6)
National priority and opportunity costs for products if the study is proved a success	66 (27.5)	121 (50.4)	53 (22.1)
Community acceptance (cultural family/group consenting)	57 (23.7)	119 (49.6)	64 (26.7)
Inherent vulnerabilities about informed consent in the local context (languages, assessment understanding, participant criteria suitability)	54 (22.5)	104 (43.3)	82 (34.2)

The level of concern was compared by types of respondents (S1 Table). More clinical researchers (34.2%) compared to non-clinical researchers (25.6%) rated as very important sponsorship and intellectual property rights (p-value = 0.01). More researchers with REC experience (46.1%) than researchers without REC experience (29.9%) rated as very important appropriate compensation for risk-taking (p-value = 0.04). More researchers with REC experience (38.2%) than researchers without REC experience (22.6%) rated as very important national priority and opportunity costs for products if the study is successful (p-value = 0.03).

## Discussion

Recently a number of reports and publications have called for greater involvement of LMICs in the conduct of human challenge studies. At the same time, worries have been raised about the ethics of these studies. Ethics frameworks for human challenge studies conducted in the US and Europe focus on ensuring the study is valuable, the risks are not excessive, and subjects give valid consent. A number of commentators have argued that these issues, although important, are not sufficient to ensure the ethical conduct of human challenge studies in LMICs. They argue that human challenge studies in LMICs need to satisfy a number of additional conditions. These include: community engagement, publicly available rationale, appropriate governance, appropriate data sharing, and compensation for research harms.

To our knowledge, this is the first study that provides empirical data regarding the attitudes among key stakeholders to human challenge studies in LMICs. As we anticipated, most respondents rated each of the 25 items as very important or important. Our discussion thus focuses on ratings of 5 (very important) as indicating the priorities of the respondents. These findings are important in several respects.

First, our study confirms the importance of the concerns that have been cited for human challenge studies in high-income countries: scientific rationale, safety, appropriate risks, and robust informed consent process. Specifically, more than 80% of respondents rated these considerations as very important. In contrast, the additional issues that have been described as important for human challenge studies in LMICs were rated as having lower importance. In particular, a publicly available rationale, national priority, and community engagement were rated as being less important. These results suggest that, in the view of important stakeholders in Thailand, the ethical issues raised by human challenge studies in LMICS do not differ significantly from the ethical issues raised by human challenge studies in high income countries [2,3,6,17,18]. Human challenge studies with promising results from high-income settings might require replications in LMICs due to the differences in host-pathogen relationships and/or disease epidemiology. The World Health Organization has noted that human challenge trials have been, and can be, successfully conducted in low- and middle-income settings by applying the same standards as in more developed countries [2]. While the conduct of challenge trials in LMICs often involves higher levels of vulnerability, the study design should employ “equivalent international” standards, including key ethical issues of acceptable levels of risk and adequately informed consent [3], which were major concerns of the participants in this study.

We found no statistically significant differences in these responses between respondents working primarily in clinical research vs. those working in nonclinical research. Similarly, we found that researchers and REC members had similar views, with the exception of risk-benefit balance and selection of study participants; i.e., more REC members than researchers rated very important for these two issues. A previous study among similar groups of researchers and ethics committee members in Thailand explored their opinions regarding ethical considerations of the research proposal and the informed-consent process in research in general, not particularly to challenge studies [19]. The results of that study identified more discrepancies of opinions between the two groups, including risk/benefit, vulnerability, confidentiality/privacy, communication of risks, decision-making authority for consent, process for approaching study participants, and the availability of a contact for study deviations/violations [19]. The present study suggests that the perceptions and concerns about challenge studies are general among researchers, and do not differ that much by research field or with experience as an REC member.

The issues that our respondents rated as most important for human challenge studies in Thailand also provide perspective on recent proposals in the literature. Worry about quality assurance is on top of the list, whereas worries about “inherent vulnerabilities about informed consent in the local context” are lowest on the list. This suggests that concerns regarding the vulnerability of participants in LMICs are not shared by important stakeholders in Thailand.

As recommended in the ethics frameworks for challenge studies, the study participants are required to be closely monitored for safety as well as for prevention of infection to others and the environment^1^. This study revealed the topmost important consideration about challenge studies in the view of researchers in Thailand is safety; about 90% of respondents in this study rated safety as very important. This finding reflects common concern among researchers in LMICs as well as those in high income countries regarding safety as the major ethical principle in designing and conducting challenge studies or other types of clinical trials.

Similarly, in contrast to the existing literature [1,6], respondents did not regard a potential lack of community acceptance as an important obstacle to human challenge studies in Thailand. This finding contrasts with a report of lessons learnt from a malaria infection study in Kenya. In that study, the researchers emphasized the importance of public engagement in promoting community perceptions of risk, disease severity, treatment availability and the baseline knowledge about the vaccines used in designing the study [12,17].

Only about one-fourth of researchers in our sample had significant concern for the readiness of infrastructure and clinical facilities for challenge studies in Thailand. This might be due to the fact there are several well-equipped laboratories and facilities and a well-established health care system in the country. These issues may be of greater concern in other LMICs [1,3,16].

Commentators have argued that human challenge studies should be conducted in LMICs only when they are a priority for the community [3,20]. WHO emphasizes that challenge studies should be judged by the degree of alignment with the national research agenda and needs for the study outcomes [3,4]. However, only about half of the respondents in Thailand rated national priority as very important.

Approximately 80% of the researchers rated as very important governance of human challenge studies in Thailand. This finding suggests, consistent with existing frameworks, that researchers in Thailand regard good governance as very important, including having data monitoring and safety boards and other relevant committees, standard operating procedures for conducting the study, qualified investigators, and a high level of quality control [3,15]. As part of good governance, several frameworks endorsed a separate group to review and approve the proposal, in addition to the standard ethics committee [2,6,21]. In this study, only about 54% of researchers rated independent review as very important.

### Limitations of the study

Our study had a number of limitations. First, the overall response rate (9%) was very low based on the 240 completed returned online questionnaires against the 2,656 email invitations that were sent out. Second, we performed the online survey distributing to two batches of the potential study population: (1) the 218 participants from 38 institutes who were participating in our workshop on human research studies, and (2) the heads of the research offices at 18 university hospitals, 84 non-university hospitals, and 22 research institutes. It should also be noted that the workshop did have a session on CHIM and there was a post-conference forum focused on CHIM, but only very few participants who were also ethics committee members from the main workshop attended this forum. As it was an anonymous survey, we did not know the sources/locations of the respondents; it might be that most of the respondents among the 240 returned questionnaire were from the 218 participants who attended the workshop. This may have biased the results. Third, most of the surveys were completed on-line and the views of individuals who lacked access to the internet may differ. This concern may be minimal for the present survey, given that the target respondents were researchers who were working in academic institutes and who generally had access to the internet as an integral part of their work. Fourth, we planned the study based on the survey on issues around the themes of the workshop we arranged. We believed that we have reviewed intensively and identified most of the important issues of human challenge models and concepts found in the literature. In our questionnaire, we defined the terminology of each item based on definitions found in the literature and attempted to rephrase it into simple language which was edited by a native English-speaking reviewer. We attempted to assess the perspectives on our key issues of interest among our target population. Thus the questionnaire might have been too specific and introduced information bias. Our study is limited to the views of professional stakeholders in Thailand. The view of the general public in Thailand and the views of professional stakeholders in other LMICs may differ. Finally, our study focused on the perspectives of stakeholders, including researchers and ethics committee members, but not members of the general community. In fact, there will be a CHIM study, funded by the Welcome Trust, on malaria vaccine in late 2019 and the researchers in that trial have been working on community engagement. The results of that study on community attitudes will complement the present study, which has presented only the viewpoints of researchers and ethics committee members.

## Conclusions

It is widely agreed that human challenge studies should satisfy a number of important ethical considerations. In addition, some commentators have argued that human challenge studies conducted in LMICs raise additional important ethical concerns. These include the importance of obtaining community input and ensuring the studies reflect national priorities. However, the respondents in the present survey, researchers and REC members in Thailand, do not appear to share these concerns. Instead, we found wide agreement on the ranking of the importance of ethical issues in our sample, consisting of both clinicians and non-clinicians, researchers and REC members. The fact that we have found a consistent pattern across a diversity of backgrounds, strengthens the conclusions.

This study reveals perceptions and concerns about challenge studies among researchers in Thailand. The researchers in clinical study and non-clinical research fields were not different in their opinions regarding the core considerations in designing and conducting the challenge studies. The researchers and the REC members also hold similar opinions regarding those core considerations, except a few issues. In general, the researchers in Thailand regard as most important: safety, informed consent process, scientific rationale, risks and harms, governance and balance of risk-benefit, respectively. If challenge studies are conducted in Thailand, major concerns that should be taken into consideration were: quality assurance procedures, appropriate compensations, community perceptions, sponsorship and intellectual propriety rights, and cost of the finished product.

## Supporting information

S1 File Survey on critical and controversial issues in health research. (PDF)

S2 File Data of characteristics of study respondents. (XLSX)

S1 Table Opinions regarding conducing challenge studies in Thailand by clinical and non-clinical researchers; Opinions regarding conducing challenge studies in Thailand by clinical and non-clinical researchers by researchers and REC members. (DOCX)
