# Supplementary material for: Conducting human challenge studies in LMICs: A survey of researchers and ethics committee members in Thailand
Source: PLoS One. 2019 Oct 10;14(10):e0223619. doi: 10.1371/journal.pone.0223619 (PMC6786649; doi:10.1371/journal.pone.0223619)
Supplement: S1 File — (PDF) [file pone.0223619.s001.pdf]

แบบสอบถามความเห็นเกี่ยวกับประเด็นเชิงวิพากษ์ด้านการวิจัยทางสุขภาพ

Survey on Critical and Controversial Issues in Health Research

แบบสอบถามนี้ มีวัตถุประสงค์เพื่อสำรวจความคิดเห็นของนักวิจัยด้านชีวการแพทย์ สาธารณสุข สังคมศาสตร์ พฤติกรรมศาสตร์ ในประเด็นที่ยังคงเป็นข้อวิพากษ์กันอยู่ในปัจจุบัน คือ การทดลองโดยนำเชื้อเข้าสู่มนุษย์ **(human challenge study)** โปรดตอบแบบสอบถามนี้ เพื่อคณะผู้จัดทำจะสามารถวิเคราะห์ความคิดเห็นของท่านต่อประเด็นเหล่านี้ เป็นการแลกเปลี่ยนเรียนรู้ และเป็นประโยชน์ต่อวงการวิชาการต่อไป อย่างไรก็ดี ท่านสามารถข้ามข้อที่ไม่ต้องการตอบโดยที่ไม่มีข้อผูกมัดใดๆ และขอรับรองว่าข้อมูลจากแบบสอบถามของท่านจะเป็นความลับและไม่สามารถระบุได้ว่าใครเป็นผู้ตอบ

ขอขอบพระคุณท่านเป็นอย่างสูงสำหรับความร่วมมือ

This survey aims to examine the opinions of researchers in the biomedical, public health and social science/behavioral sciences about current critical but controversial issues. This questionnaire is focused on **human challenge study** topic. Please complete the questionnaire to help us understand researchers' opinions on these matters. Please skip any item you do not wish to answer. This survey is guaranteed anonymous.

Thank you very much in advance for your kind responses.

ข้อมูลพื้นฐาน **(Demographic data)**

เพศ: _____ ชาย _____ หญิง
Sex: ___ Male ___ Female

ลักษณะงานวิจัยหลักของท่าน:
Main research field:
___ งานวิจัยทางคลินิก (Clinical study)
___ งานวิจัยทางชีวการแพทย์ / ห้องปฏิบัติการวิจัย (Biomedical/Laboratory Study)
___ งานวิจัยทางสาธารณสุข / งานวิจัยเชิงนโยบาย (Public Health/Policy Research)
___ งานวิจัยทางสังคมศาสตร์ / งานวิจัยทางพฤติกรรมศาสตร์ (Social Science/Behavioral Research)
___ อื่นๆ โปรดระบุ (Other please specify) ____________________

จำนวนปีที่ทำงาน: ______1-3 ปี ___ 4-6 ปี ___ 7-10 ปี ___ 11-15 ปี ___ มากกว่า 15 ปี
Years working in research field:
____ 1-3 years ____ 4-6 years ____ 7-10 years ____ 11-15 years ____ more than 15 years

ท่านดำรงตำแหน่งเป็นคณะกรรมการใดหรือไม่ (สามารถตอบได้มากกว่าหนึ่งข้อ):
Have you ever been serving as committee member? (please check all that apply):
___ ไม่เคย (No, never)
___ เป็นกรรมการจริยธรรมการวิจัยในคน (Yes, as Human Ethics Committee)
___ เป็นกรรมการจริยธรรมการวิจัยในสัตว์ (Yes, as Animal Ethics Committee)
___ เป็นกรรมการความปลอดภัยทางชีวภาพ (Yes, as Biosafety Committee)

ประเด็นเรื่อง การทดลองโดยนำเชื้อเข้าสู่มนุษย์ **(Human Challenge Study)**

คำจำกัดความ:

Definitions:

การทดลองโดยนำเชื้อเข้าสู่มนุษย์ (human challenge study หรือ controlled human infection model (CHIM)) คือการใช้เชื้อก่อโรคที่ทราบสายพันธ์ชัดเจน (well-characterized strains of an infectious agent) หรือใช้ส่วนประกอบของเชื้อในขนาดที่กำหนด (controlled doses) ผ่านวิธีการที่จำเพาะ (specific route) เพื่อให้แก่อาสาสมัครที่ผ่านการเลือกเฟ้นมาอย่างดี แล้วจึงติดตามภาวะการติดเชื้อของอาสาสมัครนี้อย่างใกล้ชิด เพื่อรักษาหรือป้องกันโรคนั้นๆ รูปแบบของการศึกษานี้ มีหลายลักษณะ เช่น 1) ทดลองแบบกระตุ้นให้เกิดโรค (challenge study) เป็นการให้เชื้อก่อโรคเข้าสู่ร่างกายอาสาสมัครที่มีสุขภาพดี 2) ทดลองแบบกระตุ้นซ้ำ (re-challenge study) เป็นการให้อาสาสมัครที่มีสุขภาพดีซึ่งเคยได้รับเชื้อก่อโรคแล้ว ได้รับเชื้อเดิม หรือสายพันธุ์ต่างกัน (heterologous strain) 3) ทดลองวัคซีนด้วยการกระตุ้นให้ติดเชื้อ (vaccine trial using a challenge model) เป็นการให้อาสาสมัครที่มีสุขภาพดีได้รับวัคซีน แล้วจึงกระตุ้นด้วยสายพันธุ์ของเชื้อก่อโรค (ในบาง การทดลอง จะกระตุ้นก่อนได้รับวัคซีน)

A human challenge study or controlled human infection model (CHIM) involves the study of well-characterized strains of an infectious agent that are administered in controlled doses and by a specific route to carefully selected adult volunteers who are closely monitored for evidence of carriage or infection under medical supervision, to anticipate or manage symptoms of disease. Study designs include: (1) challenge study, when a challenge strain of a pathogen is administered to healthy adult volunteers, (2) re-challenge study, when a challenge strain of a pathogen is administered to healthy adult volunteers previously challenged with the same strain or a heterologous strain, or (3) vaccine trial using a challenge model, when a candidate vaccine is administered to healthy adult volunteers who are later (or in some cases, previously) challenged with one or more strains of the pathogen against which the vaccine or drug is directed.

วัตถุประสงค์หลักและประโยชน์ที่คาดว่าจะได้รับจากการศึกษาแบบนี้ คือ การเร่งให้เกิดการพัฒนาวัคซีน และ/หรือ การรักษา มีประสิทธิภาพเร็วขึ้น อันจะส่งผลกระทบต่อภาระทางด้านสุขภาพของประเทศ (national health burden)

The main purpose and potential benefits of human challenge studies is to accelerate or streamline vaccines/treatment relevant to the national health burden.

วิธีตอบคำถาม:

Instructions:

โปรดให้คะแนนความสำคัญของหัวข้อต่างๆ ต่อไปนี้ (คะแนนความสำคัญเรียงลำดับจากน้อยไปมาก คือ 1 = สำคัญน้อยที่สุด / 2 = สำคัญน้อย / 3 = สำคัญ / 4 = สำคัญมากที่สุด)

Please rate the importance levels of the following items. [1=less important, 2=somewhat important 3=important, 4=very important]

หัวข้อ	ปัจจัยหลักในการพิจารณา	1	2	3	4
หลักการทางวิทยาศาสตร์<br>(Scientific rationale)	ความรู้ที่จะได้รับจากงานวิจัย มีหลักฐานชัดเจน และมีงานวิจัยรองรับ เกี่ยวกับเชื้อที่จะนำมาศึกษา<br>Knowledge to be gained from the research. Existing evidence is clearly reviewed and clearly defined				
การไม่มีทางเลือกอื่น<br>(Absence of alternative)	โจทย์วิจัยไม่สามารถจะตอบได้ด้วยการวิธีทดสอบอื่น หรือใช้เซลล์ไลน์อื่น (cell lines) เนื้อเยื่อนอกร่างกาย และสัตว์ทดลอง การทดลองโดยนำเชื้อเข้าสู่มนุษย์เป็นวิธีการเดียวที่จะใช้ในการตอบโจทย์วิจัย				

หัวข้อ	ปัจจัยหลักในการพิจารณา	1	2	3	4
	Research cannot be answered with other methods, cell lines (tissue and animal models). The challenge study is required to answer the research question.				
ปัจจัยทางเทคนิค<br>(Technical considerations)	การเลือกใช้เชื้อที่จะนำมาศึกษา และการมีอยู่ของเชื้อก่อโรคที่นำมาศึกษา ควรจะเป็นสายพันธุ์ที่เป็นปัญหาของท้องถิ่น<br>ต้องมีคำจำกัดความที่ชัดเจนของผลลัพธ์หลักที่เป็นเป้าหมายของการศึกษา (endpoints)<br>The choice and availability of a pathogen strain to be employed in the study fits with the local setting. Endpoints for CHIM studies must be clearly defined				
การตรวจสอบอย่างอิสระ<br>(Independent review)	สำหรับการศึกษา CHIM นั้น ควรจัดให้มีการตรวจสอบและการอนุมัติจากผู้เชี่ยวชาญอิสระ ที่นอกเหนือไปจากคณะกรรมการจริยธรรมการวิจัยในคน<br>For a new challenge model, the study should be reviewed and approved by independent experts in the field, in addition to a research ethics committee				
การขอความยินยอม<br>(Informed consent)	อาสาสมัครต้องรับทราบและเข้าใจเกี่ยวกับวัตถุประสงค์ วิธีการ ประโยชน์ และความเสี่ยงของการศึกษาเป็นอย่างดี<br>The participants must be well informed and understand the study’s purpose, procedures, benefits and risks				
ความปลอดภัย<br>(Safety)	มีข้อมูลที่ชัดเจนในด้านความปลอดภัย<br>Safety has been demonstrated				
สมดุลระหว่างความเสี่ยงกับประโยชน์ที่ได้รับ (Balance of risk-benefit)	ความเสี่ยงที่เกิดจากการวิจัย ต้องอยู่ในระดับที่ยอมรับได้ โดยสามารถตอบโจทย์วิจัย ทางวิทยาศาสตร์ และสมดุลกับผลประโยชน์ที่จะเกิดขึ้นในระดับชุมชน และ/หรือ ส่วนบุคคล<br>Risks are minimized consistent with the scientific ends of the study and are acceptable considering the benefits to society and/or the individual				
ความเสี่ยงและอันตราย<br>(Risks and harms)	มีการรับรองว่า อาสาสมัครจะไม่เผชิญกับความเสี่ยงที่ไม่สามารถเปลี่ยนกลับสู่สภาพปกติได้ หรือไม่สามารถรักษาได้ หรือติดเชื้อจนถึงแก่ชีวิต<br>Assurance that volunteers will never be exposed to the risks of irreversible, incurable or possibly fatal infections				
การคัดเลือกอาสาสมัคร<br>(Selection of study participants)	กระบวนการคัดเลือกอาสาสมัคร ต้องคำนึงถึงประเด็นความเปราะบางของอาสาสมัคร<br>The study recruitment procedures will protect vulnerable individuals				
ค่าชดเชยต่ออันตราย<br>(Compensation for harm)	ค่าชดเชยที่มอบให้กับอาสาสมัคร จะถูกตรวจสอบและอนุมัติโดยคณะกรรมการจริยธรรมการวิจัยในคน และต้องมีมาตรการเยียวยา อาสาสมัครในกรณีที่เกิดการบาดเจ็บที่เนื่องมาจากการศึกษา<br>Participant financial compensation will be reviewed and approved by a research ethics committee. Measures in place to compensate volunteers in the event of research-related injury				

หัวข้อ	ปัจจัยหลักในการพิจารณา	1	2	3	4
เหตุผลของการดำเนินการต้องเปิดเผยต่อสาธารณะได้ (Publicly available rationale)	มีการเปิดเผยต่อสาธารณะเกี่ยวกับประโยชน์ และความเสี่ยง การที่ไม่มีวิธีทางเลือกอื่น ตลอดจนมาตรการป้องกันอาสาสมัครและชุมชนให้ปลอดภัย The study has a clear published rationale explaining the benefits and risks, the inadequacy of alternatives and the adequacy of the measures to protect participants and the community from harm.				
มาตรการการปกป้องสาธารณะ (Protection of the public)	มีวิธีป้องกันความเสี่ยง ให้กับผู้ที่สัมผัสกับอาสาสมัคร และมีแผนการที่จะบริหารจัดการ / ควบคุม ความเสี่ยงต่อสาธารณชน There are protections for risks to others who come into contact with volunteers. The plan for public risk is well-managed/mitigated.				
ความรู้และการแบ่งปันข้อมูล (Knowledge and data sharing)	ผลจากการศึกษาสามารถก่อให้เกิดความร่วมมือ ในการพัฒนาศักยภาพทางวิทยาศาสตร์, ข้อกฎหมาย, และจริยธรรม และมีการแบ่งปันข้อมูลกันภายในชุมชนนักปฏิบัติ (community of practice) เพื่อนำไปสู่การปฏิบัติที่ดี (best practices) The study results can promote collaboration and strengthen capacity (scientific, regulatory, ethics). The data can be shared among the ‘community of practice’ to develop best practices.				
การมีส่วนร่วมของชุมชน (Community engagement)	นอกเหนือไปจากกระบวนการขอความยินยอมแล้ว การศึกษาจะต้องก่อให้เกิดความเชื่อถือต่ออาสาสมัคร ชุมชน และผู้มีส่วนได้ส่วนเสียทั้งหมด In addition to the informed consent process, the study offers trust building: participant communities and other stakeholders.				
ธรรมาภิบาล (Governance)	นักวิจัยมีธรรมาภิบาลในการทำวิจัย และมีมาตรฐานทางวิทยาศาสตร์และจริยธรรม There is a governance structure in place. There are standards in place to promote high scientific and ethical practices.				
ระดับความสำคัญของชาติ (National priority)	การศึกษาสอดคล้องกับปัญหาที่มีความสำคัญระดับชาติ (ในวาระเรื่องการวิจัย) The study corresponds with an issue of national importance (within the research agenda)				

ในความคิดเห็นของท่าน ท่านจะให้คะแนนเกณฑ์ระดับอุปสรรคหรือปัญหาในการศึกษา CHIM ในประเทศไทย อย่างไร (คะแนนเกณฑ์ระดับอุปสรรค เรียงจากน้อยไปมาก คือ 1 = ไม่มีอุปสรรคเลย 2 = มีอุปสรรคอยู่บ้าง 3 = มีอุปสรรคมาก 4 = มีอุปสรรคมากที่สุด)

In your opinion, what would be the levels of difficulty of challenges in conducting CHIM in Thailand? [1=none, 2=somewhat problematic, 3=problematic, 4=highly problematic]

อุปสรรคหรือปัญหาในการศึกษา CHIM<br>**Difficulties or Challenges in Conducting CHIM**	1	2	3	4
ความพร้อมของโครงสร้างทางกายภาพและสิ่งอำนวยความสะดวกทางการแพทย์สำหรับการศึกษา CHIM Readiness of infrastructure and clinical facilities for CHIM				
ประเด็นความยากในการขอความยินยอม เช่น การใช้ภาษาท้องถิ่น, การประเมินความเข้าใจ, ความเหมาะสมของหลักเกณฑ์ในการรับอาสาสมัคร				

อุปสรรคหรือปัญหาในการศึกษา **CHIM**<br>**Difficulties or Challenges in Conducting CHIM**	**1**	**2**	**3**	**4**
Inherent vulnerabilities about informed consent in a local context (languages, assessment understanding, participant criteria suitability)				
การยอมรับของชุมชน โดยคำนึงถึงวัฒนธรรมของกลุ่มและครอบครัว<br>Community acceptance (cultural family/group consent)				
การรับรู้และการมีส่วนร่วมของชุมชนในเรื่องเกี่ยวกับความเสี่ยงการติดเชื้อ ความรุนแรงของโรค และการรักษาที่มีอยู่<br>Community perception and engagement with respect to the understanding of infection risk, disease severity, treatment availability				
การประกันคุณภาพ/กระบวนการควบคุมคุณภาพ ของเชื้อก่อโรคในการศึกษา CHIM ตามมาตรฐานการผลิตที่ดี<br>Quality assurance/quality control procedures for agents used in CHIM following Good Manufacturing Practice (GMP) principles				
การชดเชยที่เหมาะสมต่อความเสี่ยง<br>Appropriate compensation for risk-taking				
ลำดับความสำคัญและต้นทุนค่าเสียโอกาส (opportunity costs) หากการวิจัยประสบผลสำเร็จและสามารถมีผลประโยชน์ทางพาณิชย์<br>Priority and opportunity costs for products if the study is proved a success and has commercial interest				
ความเป็นเจ้าของลิขสิทธิ์และทรัพย์สินทางปัญญาที่เป็นผลลัพธ์จากการศึกษา<br>Sponsorship and intellectual propriety rights of the finished product				

ความคิดเห็นเพิ่มเติมเรื่องการทดลองโดยนำเชื้อเข้าสู่มนุษย์ **(Other thoughts/comments about human challenge study)**

____

"ขอขอบพระคุณที่ท่านได้สละเวลาตอบแบบสอบถามชุดนี้"

Thank you very much for your time and attention.
