# Supplementary material for: Conducting human challenge studies in LMICs: A survey of researchers and ethics committee members in Thailand
Source: PLoS One. 2019 Oct 10;14(10):e0223619. doi: 10.1371/journal.pone.0223619 (PMC6786649; doi:10.1371/journal.pone.0223619)
Supplement: Supplementary file 2 [file pone.0223619.s003.docx]

Major Concerns in Conducitng CHIM	Clinical (N=123)			Non-Clinbical (N=117)			p-value
	Most Important	Important	Less Important	Most Important	Important	Less Important	
	n (%)	n (%)	n (%)	n (%)	n (%)	n (%)	
Quality assurance / quality control procedures in the agent used in CHIM following Good Manufacturing Practice principles	59 (48.0)	40 (32.5)	24 (19.5)	45 (38.5)	42 (35.9)	30 (25.6)	0.29
Appropriate compensation for risk-taking	47 (38.2)	53 (43.1)	23 918.7)	37 (31.6)	45 (38.5)	35 929.9)	0.12
Community perception and engagement with respect to the understanding of infection risk, disease severity, treatment availability	45 (36.6)	50 (40.6)	28 (22.8)	38 (32.5)	48 (41.0)	31 (26.5)	0.73
Sponsorship and intellectual propriety rights of the finished product	42 (34.2)	55 (44.7)	26 921.1)	30 (25.6)	42 (35.9)	45 (33.5)	0.01
Readiness of infrastructure and clinical facilities for CHIM	42 (34.2)	56 (45.5)	25 (20.3)	25 (21.4)	58 (49.6)	34 (29.0)	0.06
National priority and opportunity costs for products if the study is proved a success	37 (30.1)	63 (51.2)	23 (18.7)	29 (24.8)	58 (49.6)	30 (25.6)	0.38
Community acceptance (cultural family/group consenting)	34 (27.6)	61 (49.6)	28 (22.8)	23 (19.6)	58 (49.6)	36 (30.8)	0.22
Inherent vulnerabilities about informed consent in the local context (languages, assessment understanding, participant criteria suitability)	30 (24.4)	56 (45.5)	37 (30.1)	24 (20.5)	48 (41.0)	45 (38.5)	0.38

Major Concerns in Conducitng CHIM	IRB memebrs (N=76)			Researchers (N=164)			p-value
	Most Important	Important	Less Important	Most Important	Important	Less Important	
	n (%)	n (%)	n (%)	n (%)	n (%)	n (%)	
Quality assurance / quality control procedures in the agent used in CHIM following Good Manufacturing Practice principles	37 (48.7)	22 (28.9)	17 (22.4)	67 (40.8)	60 (36.6)	37 (22.6)	0.44
Appropriate compensation for risk-taking	35 (46.1)	25 (32.9)	16 (21.0)	49 (29.9)	73 (44.5)	42 (25.6)	0.04
Community perception and engagement with respect to the understanding of infection risk, disease severity, treatment availability	25 (32.9)	30 (39.5)	21 (27.6)	58 (35.4)	68 (41.5)	38 (23.2)	0.75
Sponsorship and intellectual propriety rights of the finished product	27 (35.5)	28 (36.9)	21 (27.6)	45 (27.4)	69 (42.1)	50 (30.5)	0.44
Readiness of infrastructure and clinical facilities for CHIM	24 (31.6)	34 (44.7)	18 (23.7)	43 (26.2)	80 (48.8)	41 (25.0)	0.69
National priority and opportunity costs for products if the study is proved a success	29 (38.2)	35 (46.0)	12 (15.8)	37 (22.6)	86 (52.4)	41 (25.0)	0.03
Community acceptance (cultural family/group consenting)	19 (25.0)	37 (48.7)	20 (26.3)	38 (23.2)	82 (50.0)	44 (26.8)	0.95
Inherent vulnerabilities about informed consent in the local context (languages, assessment understanding, participant criteria suitability)	16 (21.1)	34 (44.7)	26 (34.2)	38 (23.2)	70 (42.7)	56 (34.1)	0.92
